# Environmental surveillance of *Legionella* in tourist facilities of the Balearic Islands, Spain, 2006 to 2010 and 2015 to 2018

**DOI:** 10.2807/1560-7917.ES.2022.27.21.2100769

**Published:** 2022-05-26

**Authors:** Antonio Doménech-Sánchez, Elena Laso, Clara I Berrocal, Sebastián Albertí

**Affiliations:** 1 Saniconsult Ibérica SL, Palma de Mallorca, Spain; 2 Instituto Universitario de Investigación en Ciencias de la Salud (IUNICS), Universidad de las Islas Baleares, Palma de Mallorca, Spain; 3 Instituto de Investigación Sanitaria de les Illes Balears (IdIsBa), Palma de Mallorca, Spain

**Keywords:** *Legionella*, Legionnaires’ disease, hotel, cold water, hot water, whirlpool, surveillance, tourist

## Abstract

**Background:**

Legionnaires’ disease is a respiratory illness often associated with hotels and travel. Spain is a major tourist destination and one of the European countries with most cases of Legionnaires’ disease , both community- and travel-associated. However, the prevalence of *Legionella* in tourist facilities is unknown.

**Aim:**

The present investigation aimed to survey the tourist facilities in the Balearic Islands, Spain, for *Legionella* prevalence.

**Methods:**

We visited tourist facilities in the Balearic Islands in two different periods (2006–2010 and 2015–2018) and took water samples following national and international guidelines. *Legionella* was investigated by culture methods following international standards (ISO 11731:1998).

**Results:**

We evaluated 13,472 samples from 465 facilities. Bacteria of the *Legionella* genus were detected in 65.4% of the surveyed facilities. Contamination of the facilities was significantly higher during the second decade (54.5 vs 78.6%). The most frequent colonisers were *L. pneumophila* serogroup 2–14. We detected the pathogen in 15.9% and 6.9% of hot and cold water distribution systems samples, respectively. The *Legionella* contamination rate in cold water systems samples was higher when free chlorine levels were < 0.2 mg/L and at > 25 °C temperatures, while in the hot water systems samples, the contamination rate was higher at < 50 °C. Of the samples from hot tubs, 10.9% were contaminated.

**Conclusion:**

*Legionella* prevalence in hotels in the Balearic Islands was high but the contamination rates depended on the installations. Corrective measures are still needed to improve *Legionella* control.

## Introduction

*Legionella* spp. is a ubiquitous aquatic bacterium present in environmental freshwater as part of the normal flora. The genus *Legionella* consists of ca 60 species which have been isolated from aqueous environments. Half of them may infect people, primarily in the lower respiratory tract [1], causing two forms of legionellosis: Pontiac fever and Legionnaires’ disease (LD). Pontiac fever is thought to constitute ca 95% of cases and is a self-limiting illness with influenza-like symptoms, while LD consists of atypical pneumonia with symptoms ranging from mild illness to severe pneumonia. The incubation period is 2–14 days, and the severity of symptoms is related to age and immunodeficiencies, although male sex and smoking are also recognised risk factors. The mortality rate of LD is 10–15%, and the higher rates relate to infections that occur in healthcare facilities. Several species of *Legionella* may cause LD pneumonia, and *L. pneumophila* is the most frequently associated [1]. This species classifies into different serogroups, serogroup 1 being the most prevalent among cases. This air-borne pathogen transmits in small droplets known as aerosols, which allow the bacterium to reach the alveoli.

*Legionella* colonises man-made facilities such as water distribution systems (WDS) and cooling towers located in hospitals, hotels and other public and industry buildings, and all these facilities have been associated with LD [2-5]. Most cases of LD are community-acquired (67%), followed by travel-related (24%) and healthcare-related cases (5%) [1,6,7]. In the European Union and European Economic Area, 28 countries reported 11,298 LD cases in 2019 [6]. This represents the highest rate of notification in the historical data. Three out of four notified cases were reported by France, Germany, Italy and Spain [6]. Travel-associated Legionnaires’ disease (TALD) stands for LD cases of travellers who acquire the infection in the country of visit but, because of the long incubation period, may develop the symptoms and be diagnosed at home. The number of TALD was 1,657 in 2019, again the highest figures ever observed. Most of the cases were associated with visits to Italy, France and Spain [6], and patients had stayed in hotels in 75% of cases. Altogether, this indicates need to understand the epidemiology of LD to improve risk evaluation, detection of pathogen niches and investigation and control of cases and outbreaks, particularly in hotels [1].

Spain is a major Mediterranean tourist destination. Prevalence of *Legionella* in hotels in similar destinations (Greece, Italy, Turkey) has been widely investigated [8-14], but information from Spanish tourist facilities remains scarce. These accommodations have recirculating water systems with storage tanks, which increases the risk of legionellosis [8]. The only data available at the time of our study came from one study of 231 samples limited to the hot water system [15]. Recently, another manuscript on *Legionella* in hotels located in the Canary Islands has been published [16]. The present investigation aimed to survey the tourist facilities in the Balearic Islands, Spain, for *Legionella* prevalence. The objective was to get a representative picture of the situation, including several installation types during two different periods (2006–2010 and 2015–2018). This knowledge will allow the design of appropriate improvement measures to reduce the risk associated with these facilities.

## Methods

### Tourist facilities

The tourist facilities surveyed in the present investigation included hotels, apartments and agritourism resorts located in the Balearic Islands, Spain. We visited 465 facilities, representing 32% of tourist facilities in our region, in two different periods: from 10 January 2006 to 21 October 2010 and from 12 January 2015 to 27 December 2018. They included 319 and 210 facilities, respectively, with 63 hotels participating in both periods. Visits were unannounced in order to observe the situation in routine working conditions. Hotels were visited on average six times.

### Sampling procedure

The sampling points were selected based on the characteristics of the facilities, following the recommendations of the Spanish Ministry of Health [17,18]. They included hot and cold WDS and pools with jets, waterfalls and/or air bubblers such as hot tubs). The presence of cooling towers in hotels in our region is negligible, so they were ignored. Water samples from bathroom outlets (showerheads or bath taps) were collected without flaming the outlet point and by the pre-flush technique, i.e. without letting the water run beforehand. This represents the best simulation for common use conditions and user exposure. Following national and international recommendations [18,19], samples of 1 L were collected into sterile bottles containing 20 mg sodium thiosulphate (Sharlab, Spain), able to neutralise up to 5 mg/L free and combined chlorine. The temperature was determined in situ with a calibrated digital Testo 104 thermometer (Testo, Spain) one minute after flushing. Free chlorine levels in cold water were also determined in situ with the Lovibond portable MD100 instrument (Lovibond, Germany) by the colorimetric method described in [20]. Cold samples were transported in refrigeration whereas hot samples were transported at room temperature. All samples arrived at the laboratory less than 8 h after sampling and were processed immediately or stored in refrigeration.

### Definition of disinfectant and temperature ranges 

Water disinfection (mainly with chlorine derivatives) is a crucial strategy for *Legionella* control in cold WDS. In Spain, the legislation for the prevention of Legionnaires’ disease in cold water establishes that chlorine levels should not drop below 0.2 mg/L in the WDS [18]. In addition, drinking water legislation restricts the levels of this disinfectant to a maximum of 1 mg/L [21]. According to these values, we defined three groups of free chlorine levels (< 0.2, 0.2–1 and > 1 mg/L) to investigate the relationship between disinfectant levels and *Legionella* contamination.

Temperature is another key factor to prevent *Legionella* contamination [22]. In fact, it is the main factor for *Legionella* eradication in hot WDS systems where chlorine compounds are volatile. Hot water temperature should not drop below 50 °C in any part of the system at any time, whereas storing water at 60 °C or higher is recommended [17,18]. We defined three ranges of temperature to study the relationship between temperature and presence of *Legionella* in hot water: < 50 °C, 50–60 °C and > 60 °C. 

### Laboratory investigation

The procedure for *Legionella* detection and enumeration in the water samples was based on international standards [23]. *Legionella* from 1 L of the sample were concentrated using a 47 mm nitrocellulose membrane with 0.22 µm pores (Sartorius SA, Spain). After filtration, the membrane was aseptically placed into one screw-capped sterile tube containing 10 mL sample. Bacterial cells were dislodged from the membrane by vortex for at least 2 min. Two 0.5 mL aliquots were directly plated onto glycine, vancomycin, polymyxin and colimicyn (GVPC) medium plates (Oxoid, Spain). To reduce the number of interfering bacteria, 1 mL from the tube was acid-treated with 0.2 mol 1:1 HCl-KCl at pH 2.2 for 5 min. Another mL was heat-treated at 50 °C in a water bath for 30 min. After the treatments, 0.1 mL were inoculated onto GVPC plates. All plates were incubated at 36 ± 1 °C for 10 days in anaerobic jars containing CO_2_Gen Compact sachets (Oxoid, Spain) to generate a 2.5–5% CO_2_ atmosphere. Readings were performed on days 4, 7 and 10. Colonies with characteristic morphological features compatible with *Legionella* detected in any GVPC plate were considered as presumptive *Legionella*. For confirmation, at least three of them were selected and subcultured in buffered charcoal yeast extract (BCYE) and BCYE without cysteine (BCYE-cys) media (Oxoid, Spain). Isolates growing on BCYE but not on BCYE-cys were considered as members of the *Legionella* genus. The commercially available *Legionella* latex agglutination test (Oxoid, Spain) was used for serotyping, identifying the isolates as *L. pneumophila* serogroup 1, *L. pneumophila* serogroup 2–14 and *Legionella* non-*pneumophila* species. The detection limit of the procedure was 10 colony-forming units (CFU)/L. The conventional ranges for minimal (< 3 log CFU/L), moderate (3–4 log CFU/L) and high contamination (> 4 log CFU/L) were applied to the positive samples [17,23,24].

### Data analysis

The collected data from the study were imported into a Microsoft Excel 2016 file from the Laboratory Integrated Management System (LIMS). Data were curated by analysing the metadata of the samples (hotel, sample code, date, installation etc) to detect and eliminate duplicates and inconsistencies, and we finally considered values for 13,472 samples. The CFU/L values were converted into log CFU/L values before analysis. We used the two-tailed chi-squared test for the qualitative data analysis (*Legionella* presence/absence, temperature or chlorine ranges, type of installation etc) and the t-test for quantitative data (bacterial counts). Results were considered statistically significant at p values < 0.05. All statistical analyses were also performed in Microsoft Excel 2016.

## Results

### *Legionella* contamination in tourist facilities in the Balearic Islands

The extent and characteristics of *Legionella* contamination in tourist facilities are shown in Table 1. Overall, 13,472 samples from 465 tourist facilities were collected and analysed during the study (mean: 29 samples/hotel; range: 1–507; median: 19 samples/hotel). The presence of *Legionella* was detected in 65.4% of the surveyed hotels (304/465), with a significant intensification of the facilities contamination during the second period (54.5%; 174/319 vs 78.6%; 165/210). *L. pneumophila* was the predominant species in both periods irrespective of whether the hotels were contaminated by one or several species or serogroups. Serogroup 2–14 were the most frequent colonisers, followed by *L. pneumophila* serogroup 1. The bacterial load in the contaminated hotels was higher in the first period. All these features were also observed when only the subset of hotels visited in both periods were considered (see Supplementary Table S1 for this sensitivity analysis).

**Table 1:** Characteristics of *Legionella* contamination in the investigated facilities, Balearic Islands, Spain, 2006–2010 and 2015–2018 (n = 465 facilities)

Parameter	Entire study				First period (2006–2010)				Second period (2015–2018)			
	n	%	log CFU/L		n	%	log CFU/L		n	%	log CFU/L	
			Mean	Range			Mean	Range			Mean	Range
Hotels^a^	n = 465				n = 319				n = 210			
Not contaminated by Lspp	161	34.6	NA		145	45.5	NA		45	21.4	NA	
Contaminated by Lspp	304	65.4	2.68	0.5–5.6	174	54.5	2.84	1.3–4.9	165	78.6	2.51	0.5–5.6
One single species/serogroup	153	32.9	2.65	0.5–5.3	122	38.2	2.84	1.3–4.9	56	26.7	2.53	0.5–5.6
LP1	50	10.8	2.83	0.9–5.0	39	12.2	2.86	1.3–4.7	17	8.1	2.54	0.5–5.1
LP2–14	93	20.0	2.75	0.5–5.3	75	23.5	2.85	1.3–4.9	37	17.6	2.49	0.5–5.6
LnP	10	2.2	2.17	1.3–4.6	8	2.5	2.64	1.3–4.9	2	1.0	2.58	0.5–5.0
Several species/serogroups	151	32.5	2.74	0.5–5.6	52	16.3	2.82	1.3–4.9	109	51.9	2.54	0.5–5.6
LP1 + LP2–14	76	16.3	2.56	0.5–5.6	20	6.3	2.77	1.3–4.9	61	29.0	2.49	0.5–5.6
LP1 + LnP	10	2.2	2.77	1.3–4.6	9	2.8	2.81	1.3–4.7	4	1.9	2.63	1.3–4.0
LP2–14 + LnP	22	4.7	2.84	0.5–4.7	16	5.0	2.78	1.3–4.7	10	4.8	2.58	0.5–4.6
LP1 + LP2–14 + LnP	43	9.2	2.68	0.5–5.1	7	2.2	3.04	1.3–4.9	34	16.2	2.58	0.5–5.1
Samples	n = 13,472				n = 7,113				n = 6,359			
Not contaminated by Lspp	11,807	87.6	NA		6,311	88.7	NA		5,496	86.4	NA	
Contaminated by Lspp	1,665^b^	12.4	2.67	0.5–5.6	802^b^	11.3	2.84	1.3–4.9	863	13.6	2.51	0.5–5.6
LP1	567	4.2	2.70	0.5–5.1	269	3.8	2.87	1.3–4.7	298	4.7	2.54	0.5–5.1
LP2–14	966	7.2	2.70	0.5–5.6	476	6.7	2.84	1.3–4.9	490	7.7	2.49	0.5–5.6
LnP	138	1.0	2.60	0.5–5.0	63	0.9	2.70	1.3–4.9	75	1.2	2.58	0.5–5.0

Lspp: bacteria of the genus *Legionella;* LP1: *Legionella pneumophila* serovar 1; LP2–14: *L. pneumophila* serovar 2–14; LnP: *Legionella* non-*pneumophila* ; NA: not applicable.

^a^ Sixty-three hotels were visited in both periods.

^b^ Six samples were contaminated by *Legionella* from two different groups.

Consistent with this result, 12.4% of the samples (1,665/13,472) were positive for *Legionella*, with a slight increase in the second period (11.3%; 802/7,113 vs 13.6%; 863/6,359). *L. pneumophila* was again the predominant species in both periods, being the serogroup 2–14 the most frequently isolated, followed by *L. pneumophila* serogroup 1. Moreover, the bacterial load in the positive samples was also higher in the first period, when 16.0% of samples (128/802) were highly contaminated (> 4 log CFU/L). Nevertheless, bacterial load was minimal (< 3 log CFU/L) in more than half of the samples (1,041/1,665) from both periods (data not shown).

### *Legionella* contamination in the water distribution system

We collected 11,797 samples from the WDS of 458 tourist facilities: 6,859 during the first period and 4,938 during the second one. In contrast to the previous chapter, this does not include the 1,675 samples from hot tubs. The period 2006 to 2010 included 4,438 hot WDS samples (2,261 from the circuits and 2,177 from the tanks) and the period 2015 to 2018 included 3,005 (2,084 from the circuits and 921 from the tanks). Regarding the cold WDS, 2,421 samples were collected in the first period (1,835 from the circuits and 586 from the tanks) and 1,933 in the second period (1,471 from the circuits and 462 from the tank).

The analysis of the contamination levels of the visited hotels indicated that the hot WDS of 60.0% of the hotels (272/453) were colonised by *Legionella* (Table 2). The prevalence of *Legionella* did not differ between circuits and tanks. However, the bacterial load was higher in the tanks. The complete analysis of the 7,443 samples from hot WDS included 4,345 from the circuits and 3,098 from storage tanks. The overall contamination rate was 15.9% (1,182/7,443), with no significant differences between circuits and tanks samples. Still, as observed at the hotel level, bacterial load was higher in the tanks. The rate of highly contaminated samples was also higher in those components (data not shown).

**Table 2:** Characteristics of *Legionella* contamination in the hot water distribution systems of investigated hotels, Balearic Islands, Spain, 2006–2010 and 2015–2018 (n = 453 hotels^a^)

Parameter	Hotels				Samples			
	n	%	log CFU/L		n	%	log CFU/L	
			Mean	Range			Mean	Range
Circuits								
Number	n = 448				n = 4,345			
Not contaminated by Lspp	225	50.2	NA		3,667	84.4	NA	
Contaminated by Lspp	223	49.8	2.58	0.5–5.3	678	15.6	2.58	0.5–5.3
**One single species/serogroup**	**149**	**33.3**	**2.67**	**0.5–5.3**	**675**	**15.5**	**2.58**	**0.5–5.3**
LP1	43	9.6	2.81	0.9–5.0	204	4.7	2.62	0.9–5.1
LP2–14	98	21.9	2.63	0.5–5.3	425	9.8	2.56	0.5–5.3
LnP	8	1.8	2.44	1.3–3.3	46	1.1	2.54	0.5–4.5
**Several species/serogroups**	**74**	**16.5**	**2.49**	**0.5–5.1**	**3**	**0.1**	**2.66**	**0.9–5.3**
LP1 + LP2–14	40	8.9	2.41	0.5–4.9	1	0.0	2.67	2.67
LP1 + LnP	1	0.2	2.16	2.16	0	0.0	NA	
LP2–14 + LnP	11	2.5	2.78	0.5–4.6	2	0.0	2.65	0.9–5.3
LP1 + LP2–14 + LnP	22	4.9	2.51	0.5–5.1	0	0.0	NA	
Tanks	n = 397				n = 3,098			
Not contaminated by Lspp^a^	205	51.6	NA		2,594	83.7	NA	
Contaminated by Lspp	192	48.4	3.01	0.5–5.6	504	16.3	3.01	0.5–5.6
**One single species/serogroup**	**138**	**34.8**	**3.02**	**1.3–5.6**	**501**	**16.2**	**3.01**	**0.5–5.6**
LP1	45	11.3	3.09	1.3–5.1	165	5.3	3.01	0.5–5.6
LP2–14	86	21.7	3.01	1.3–5.6	292	9.4	3.03	1.3–5.6
LnP	7	1.8	2.71	1.3–4.6	44	1.4	2.93	0.5–5.0
**Several species/serogroups**	**54**	**13.6**	**3.01**	**0.5–5.0**	**3**	**0.1**	**3.20**	**2.9–3.2**
LP1 + LP2–14	31	7.8	2.96	1.3–4.9	3	0.1	3.20	2.9–3.2
LP1 + LnP	5	1.3	3.30	0.5–4.9	0	0.0	NA	
LP2–14 + LnP	13	3.3	3.01	1.3–4.7	0	0.0	NA	
LP1 + LP2–14 + LnP	5	1.3	3.00	1.1–5.0	0	0.0	NA	
Total								
Number	n = 453				n = 7,443			
Not contaminated by Lspp^a^	181	40.0	NA		6,261	84.1	NA	
Contaminated by Lspp	272	60.0	2.76	0.5–5.6	1,182	15.9	2.76	0.5–5.6
**One single species/serogroup**	**155**	**34.2**	**2.83**	**0.5–5.3**	**1,176**	**15.8**	**2.77**	**0.5–5.6**
LP1	45	9.9	3.00	0.9–5.0	369	5.0	2.80	0.5–5.1
LP2–14	101	22.3	2.76	0.5–5.3	717	9.6	2.76	0.5–5.6
LnP	9	2.0	2.81	1.3–4.6	90	1.2	2.72	0.5–5.0
**Several species/serogroups**	**117**	**25.8**	**2.73**	**0.5–5.6**	**6**	**0.1**	**2.93**	**0.9–5.3**
LP1 + LP2–14	65	14.3	2.70	0.5–5.6	4	0.1	3.07	2.7–3.2
LP1 + LnP	6	1.3	2.96	1.3–4.6	0	0.0	NA	
LP2–14 + LnP	18	4.0	2.95	0.5–4.9	2	0.0	2.65	0.9–5.3
LP1 + LP2–14 + LnP	28	6.2	2.65	0.5–5.1	0	0.0	NA	

Lspp: bacteria of the genus *Legionella;* LP1: *Legionella pneumophila* serovar 1; LP2–14: *Legionella pneumophila* serovar 2–14; LnP: *Legionella* non-*pneumophila*.

^a^ In five of the total 458 facilities, only the cold water distribution system was sampled these are therefore not included in this analysis of the hot water systems.

We evaluated the data from the cold WDS in 448 hotels (Table 3). The cold WDS of 35.7% of the surveyed hotels (160 /448) were colonised by *Legionella*. The percentage of hotels with contaminated cold water circuits was four times higher than that of hotels with contaminated tanks (33.1 vs 7.8%). We analysed 4,354 samples comprising 3,306 from the circuits and 1,048 from the tanks, and 6.9% of the samples (300/4,354) tested positive for *Legionella*. Supporting the observations at the hotel level, and in contrast to the hot WDS samples, the colonisation in the circuits was higher than that detected in the tanks. Although the bacterial loads were again more abundant in the tanks, the differences were not so prominent. Nevertheless, the percentage of highly contaminated samples was higher in the tanks (data not shown).

**Table 3:** Characteristics of *Legionella* contamination in the cold water distribution systems of investigated hotels, Balearic Islands, Spain, 2006–2010 and 2015–2018 (n = 448 hotels)

Parameter	Hotels				Samples			
	n	%	logCFU/L		n	%	log CFU/L	
			Mean	Range			Mean	Range
Circuits								
Number	n = 447^a^				n = 3,306			
Not contaminated by Lspp	299	66.9	NA		3,037	91.9	NA	
Contaminated by Lspp	148	33.1	2.41	0.5–4.7	269	8.1	2.40	0.5–4.7
One single species/serogroup	120	26.8	2.68	0.5–5.6	268	8.1	2.42	0.5–4.7
LP1	40	8.9	2.65	0.9–5.0	102	3.1	2.50	0.9–4.7
LP2–14	67	15.0	2.70	0.5–5.6	146	4.4	2.39	0.5–4.6
LnP	13	2.9	2.70	0.5–5.1	20	0.6	2.13	1.3–3.9
Several species/serogroups	28	6.3	2.70	0.5–5.1	1	0.0	2.85	2.85
LP1 + LP2–14	22	4.9	2.70	0.5–4.9	0	0.0	NA	
LP1 + LnP	1	0.2	2.89	2.89	0	0.0	NA	
LP2–14 + LnP	4	0.9	2.65	0.8–5.1	1	0.0	2.85	2.85
LP1 + LP2–14 + LnP	1	0.2	2.62	2.62	0	0.0	NA	
Tanks								
Number	n = 344				n = 1,048			
Not contaminated by Lspp	317	92.2	NA		1,017	97.0	NA	
Contaminated by Lspp	27	7.8	2.61	0.7–4.9	31	3.0	2.62	0.5–4.9
One single species/serogroup	25	7.3	2.47	0.5–5.1	31	3.0	2.62	0.5–4.9
LP1	12	3.5	2.45	0.5–5.10	14	1.3	2.69	1.3–4.9
LP2–14	9	2.6	2.47	0.5–4.9	11	1.0	2.40	0.5–3.5
LnP	4	1.2	2.52	0.5–4.6	6	0.6	2.87	1.3–4.2
Several species/serogroups	2	0.6	3.05	2.9–3.2	0	0.0	NA	
LP1 + LP2–14	0	0.0	NA		0	0.0	NA	
LP1 + LnP	1	0.3	2.9	2.9	0	0.0	NA	
LP2–14 + LnP	1	0.3	3.2	3.2	0	0.0	NA	
LP1 + LP2–14 + LnP	0	0.0	NA		0	0.0	NA	
Total								
Number	n = 448				n = 4,354			
Not contaminated by Lspp	288	64.3	NA		4,054	93.1	NA	
Contaminated by Lspp	160	35.7	2.43	0.5–4.9	300	6.9	2.43	0.5–4.9
One single species/serogroup	126	28.1	2.63	0.5–5.6	299	6.9	2.43	0.5–4.9
LP1	45	10.0	2.58	0.5–5.0	116	2.7	2.52	0.9–4.9
LP2–14	68	15.2	2.68	0.5–5.6	157	3.6	2.39	0.5–4.6
LnP	13	2.9	2.56	0.5–4.6	26	0.6	2.30	1.3–4.2
Several species/serogroups	34	7.6	2.70	0.5–5.1	1	0.0	2.85	2.85
LP1 + LP2–14	22	4.9	2.66	0.5–4.9	0	0.0	NA	
LP1 + LnP	3	0.7	2.96	0.7–5.1	0	0.0	NA	
LP2–14 + LnP	4	0.9	2.59	0.5–4.9	1	0.0	2.85	2.85
LP1 + LP2–14 + LnP	5	1.1	2.84	1.3–5.1	0	0.0	NA	

Lspp: bacteria of the genus *Legionella;* LP1: *Legionella pneumophila* serovar 1; LP2–14: *Legionella pneumophila* serovar 2–14 ; LnP: *Legionella* non-*pneumophila*.

^a^ For some hotels not all the installations were sampled.

Finally, we compared the data from the hot and cold WDS samples. Remarkably, the rate of *Legionella* contamination in hot WDS was about two times higher than in the cold WDS, in the analysis of both hotels and samples. The comparison between circuits also showed that the colonisation rate, as well as the observed bacterial load, were higher in the hot WDS circuits than in the cold WDS circuits. When we compared the storage components, the colonisation rates were more than five times higher in hot WDS tanks, and the bacterial load was also higher. Irrespective of the type of distribution system (circuit or tank, different water temperature), bacteria belonging to serogroups 2–14 of *L. pneumophila* were always the most frequent, followed by *L. pneumophila* serogroup 1 isolates and finally, the *Legionella* non-pneumophila species.

### Relationship between *Legionella* contamination and temperature or free chlorine levels

**Figure 1:**
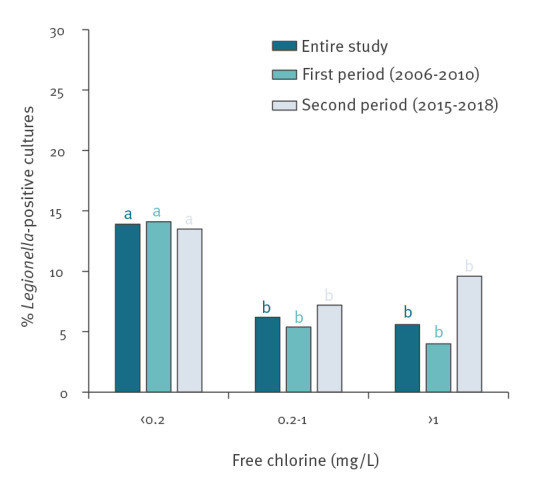
Relationship between free chlorine levels in cold water distribution systems and *Legionella* contamination, Balearic Islands, Spain, 2006–2010 and 2015–2018 (n = 4,156 samples)^a^

^a ^Chlorine values were not available for 196 cold water samples.

We defined three groups of free chlorine levels (< 0.2, 0.2–1 and > 1 mg/L) to investigate the relationship between disinfectant levels and *Legionella* contamination. The rate of contamination in the cold WDS system was dependent on chlorine levels (Figure 1). The pathogen contaminated more frequently those samples with < 0.2 free chlorine mg/L. The results were not statistically different when comparing disinfectant levels ≥ 0.2 mg/L. These observations also applied when we evaluated decades independently.

Different letters with the same colour designate significant differences among ranges in the same period (p < 0.05).

**Figure 2:**
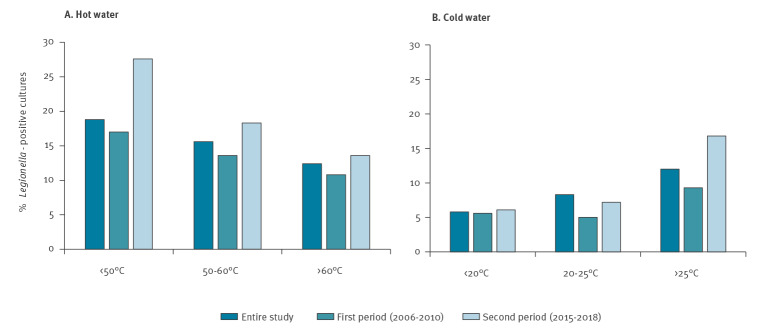
Relationship between the water temperature and *Legionella* contamination, Balearic Islands, Spain, 2006–2010 and 2015–2018 (n = 11,797 samples)

*Legionella* was more prevalent when the hot water temperature did not reach 50 °C (Figure 2). No significant differences were found between the contamination rates in the other two groups (50–60 °C and > 60 °C) in the entire study and during the first period, whereas in the period 2015 to 2018, temperatures > 60 °C were more efficient than 50–60 °C in reducing *Legionella* contamination.

In the cold WDS, the temperature must remain under 20 °C to avoid the growth of the pathogen. In Spain, the originally mandatory < 20 °C level changed to a recommended level because it was impossible to maintain during the summer season. Nevertheless, *Legionella* growth rate rises at temperatures above 25 °C, constituting a risk. Thus, as other investigators [9], we used it as a cut-off point and selected three temperature ranges at < 20 °C, 20–25 °C and > 25 °C (Figure 2). As expected, the percentage of positive cold WDS samples was larger at the highest temperatures (> 25 °C). We did not find differences between < 20 °C and 20–25 °C samples.

### *Legionella* contamination in hot tubs and spa-like facilities

Legionellosis has been associated with the use of whirlpools, hot tubs and other spa-associated accommodations. From this moment on, we will refer generically to these facilities as hot tubs. We evaluated 1,675 samples from 296 hot tubs located in 227 hotels in this survey. We detected *Legionella* in the hot tubs of 44.5% of the hotels (Table 4). Again, isolates of *L. pneumophila* were more frequently present than the other members of the genus. In this case, no differences between serogroups were apparent in the contamination of hot tubs. *Legionella* were detected in 10.9% of the samples. However, in most cases, the pathogen contamination was minimal (data not shown). As we observed in the other facilities, we detected a higher contamination rate during the second study period and a higher bacterial load in the first study period (data not shown).

**Table 4:** Characteristics of *Legionella* contamination in the hot tubs from the investigated hotels, Balearic Islands, Spain, 2006–2010 and 2015–2018 (n = 227)

Parameter	Hotels				Samples			
	n	%	logCFU/L		n	%	logCFU/L	
			Mean	Range			Mean	Range
Number	n = 227				n = 1,675			
Not contaminated by Lspp	126	55.5	NA		1,492	89.1	NA	
Contaminated by Lspp	101	44.5	2.45	0.5–4.6	183	10.9	2.45	0.5–4.6
One single species/serogroup	76	33.5	2.37	0.8–4.6	183	10.9	2.37	0.8–4.6
LP1	34	15.0	2.47	0.8–4.6	78	4.7	2.48	0.4–4.6
LP2–14	35	15.4	2.31	1.3–3.8	85	5.1	2.40	1.3–4.3
LnP	7	3.1	2.14	1.3–3.7	20	1.2	2.53	1.3–4.6
Several species/serogroups	25	11.0	2.55	0.5–4.6	0	0.0	NA	
LP1 + LP2–14	15	6.6	2.46	0.5–4.2	0	0.0	NA	
LP1 + LnP	4	1.8	2.70	2.0–3.7	0	0.0	NA	
LP2–14 + LnP	2	0.9	2.61	0.7–3.4	0	0.0	NA	
LP1 + LP2–14 + LnP	4	1.8	2.70	1.3–4.6	0	0.0	NA	

Lspp: bacteria of the genus *Legionella;* LP1: *Legionella pneumophila* serovar 1; LP2–14: *Legionella pneumophila* serovar 2–14; LnP: *Legionella* non-*pneumophila*.

## Discussion

Spain is one of the four European countries with most community-acquired LD and TALD cases [6]. However, the prevalence of *Legionella* in Spanish hotels has been poorly investigated [15]. In this study, we evaluated the prevalence of this pathogen in a large number of tourist facilities from a major tourist destination in Spain, the Balearic Islands. Members of the *Legionella* genus contaminated 65.4% of all tourist facilities evaluated in this study. This contamination rate is similar what has been reported in studies conducted in Hungary (72%) [25], Italy (66.9%) [3], Greece (75%) [9] and the Netherlands (85%) [26] but remarkably higher than rates reported in studies performed in Croatia (27.2%) [27] and Italy (35.1%) [24]. Several non-exclusive factors might explain this apparent discrepancy between studies and countries, such as the number of facilities or samples analysed in each study, the period covered and the type of installation evaluated, usually focused on hot WDS. In our study, we included a large number of facilities and samples, covered 9 years in two different periods and evaluated cold and hot WDS as well as hot tubs to strengthen our results. Furthermore, we selected touristic facilities randomly, in contrast with other studies that selected accommodations based on previous LD episodes, thereby introducing a bias. Thus, our results provide reliable figures for *Legionella* colonisation of tourist facilities, at least in our country. *Legionella pneumophila* was the most prevalent species in our community, and serogroups 2–14 were the most frequently isolated. This is in accordance with previous investigations in Europe [3,8-10,13,14,24], although *L. pneumophila* serogroup 1 was the predominant in one study in Turkey [28].

Most *Legionella* prevalence studies focus on hot WDS because they are considered high-risk installations for *Legionella* dissemination. In our study, 60% of hotels were colonised with *Legionella,* similar to observations in Greece (66.7%) [12] and Italy (63%) [10,11]. However, we detected *Legionella* only in 15.9% of the hot WDS samples, a level of contamination lower than reported in other European countries, including Greece (28%) [9], Italy (32%) [10] and Hungary (72%) [25]. We did not find any differences between the contamination rates of distal points and tanks. However, the hot WDS tanks constitute a riskier installation as they had higher bacterial loads and a higher frequency of highly contaminated samples. Therefore, specific interventions to improve these particular installations are advisable. They may include increasing the temperature and the frequency of cleaning and disinfection which currently is yearly [17]. The prevalence of *Legionella* was higher at temperatures < 50 °C. That is an expected risk, as the pathogen can resist these temperatures. There are several investigations on temperature effects in hot WDS, reviewed in [29]. Some of them describe protective effects for ≥ 60 °C, whereas other studies set the threshold at ≥ 55 °C. However, our results demonstrate that *Legionella* can colonise the hot water installations even at ≥ 60 °C temperatures, suggesting that higher temperatures are required to eradicate *Legionella* from these installations.

We detected *Legionella* in 6.9% of cold WDS samples, which is clearly lower compared with other European studies conducted in hotels (21.4%) [9] and healthcare facilities (36.3%) [30]. The prevalence and bacterial load of *Legionella* species in the cold WDS supply of our tourist facilities are undoubtedly lower than those for hot WDS, both in the circuits and the tanks. This is in agreement with the fact that cold WDS are considered low-risk installations. However, fatal cases of legionellosis have been linked to cold WDS [4], and evaluation of these installations should be routinely performed. This is especially important when risk factors favouring *Legionella* proliferation are present, such as the high environmental temperatures in Mediterranean countries in summer. Thus, Spanish guidelines include routine evaluations [17], but not the guidelines of some European countries with temperate climate such as Germany [30]. This aspect should be borne in mind given the global increase in temperatures throughout the year.

It is noteworthy that in our study, the circuits of the cold WDS presented higher colonisation rates than the tanks. In these installations, control of *Legionella* is based on disinfection and temperature. Our results demonstrate that samples with free chlorine levels below the legal limit (0.2 mg/L) are more often contaminated by the pathogen. A positive association with free chlorine < 0.375 mg/L was also found by Kyritsi et al. [9]. In the case of temperature, our results indicate that *Legionella* is more frequent in samples above 25 °C. By contrast, Greek investigators did not find such differences [9,31]. Interventions such as proper isolation preventing that the cold water is heated by high environmental temperatures or the proximity of hot WDS should be implemented to improve *Legionella* control.

Wellness centres offer hot tubs and similar bathing facilities and pose health risks related to LD. Although they are present in several tourist accommodations, they are rarely included in surveys. The contamination rate in our samples was 10.9%, clearly lower than the 50%, 75% or even 85% previously described [9,24,26]. In addition, the level of contamination detected was minimal in most cases. The risk associated with these pools in the hotels located in our community seems lower than initially expected.

We did not detect any relationship between contamination of facilities in a given year and LD cases in the Balearic Islands, including TALD. Nor did we find a seasonal trend was found for *Legionella* prevalence, as previously noted [31]. However, colonisation of hotels was higher in the second period, although bacterial levels were lower. The same was true when we analysed only those hotels investigated in both periods. In Spain, hotels must implement water safety plans with continuous monitoring of the water quality and periodic sampling of *Legionella* [17,18]. Current practices seem to reduce bacterial proliferation in the installations but are not effective in avoiding pathogen entry. This aspect should be further investigated to improve current practices.

Finally, some limitations apply to our study. The latex test used for serotyping groups many serogroups together into one category [2-14], thus missing some valuable information. The number of samples from faucets was negligible (25/7,645). Therefore, it was not possible to compare them with samples from showers. The same applies to the investigated indoor pools (63/1,675), which prevents their comparison with hot tubs. These comparisons may be useful to explain some differences in the facility positivity rates.

## Conclusion

We characterised the prevalence of *Legionella* in tourist facilities in the Balearic Islands, Spain. Different installations were extensively analysed and in most cases, the percentage and/or levels of colonisation were different. Nevertheless, further surveys in different Spanish regions are desirable, as regional variability has been described. As a result of our investigation, we recommend specific measures to improve *Legionella* control in particular installations, such as increasing the temperature of hot WDS and the frequency of cleaning and disinfection, together with isolation of cold WDS circuits.

## Supplementary Data

82000 Supplement
