# Supplementary material for: Environmental surveillance of Legionella in tourist facilities of the Balearic Islands, Spain, 2006 to 2010 and 2015 to 2018
Source: Euro Surveill. 2022 May 26;27(21):2100769. doi: 10.2807/1560-7917.ES.2022.27.21.2100769 (PMC9137269; doi:10.2807/1560-7917.ES.2022.27.21.2100769)
Supplement: Supplement [file 21-00769_DOMENECH-SANCHEZ_Supplement.pdf]

# Supplementary material for manuscript

"Environmental surveillance of Legionella in tourist facilities of the Balearic Islands, Spain, 2006 to 2010 and 2015 to 2018"

This supplementary material is hosted by *Eurosurveillance* as supporting information alongside the article "Environmental surveillance of Legionella in tourist facilities of the Balearic Islands, Spain, 2006 to 2010 and 2015 to 2018", on behalf of the authors, who remain responsible for the accuracy and appropriateness of the content. The same standards for ethics, copyright, attributions and permissions as for the article apply. Supplements are not edited by *Eurosurveillance* and the journal is not responsible for the maintenance of any links or email addresses provided therein.

**Table S1. Characteristics of *Legionella* contamination in the hotels investigated in the two periods, Balearic Islands, Spain, 2006-10 and 2015-18 (n=63)**

| Parameter | Entire study | | | | First period (2006-2010) | | | | Second period (2015-18) | | | |
|---|---|---|---|---|---|---|---|---|---|---|---|---|
| | n | % | Mean count (logCFU $L^{-1}$) | Range (logCFU $L^{-1}$) | n | % | Mean count (logCFU $L^{-1}$) | Range (logCFU $L^{-1}$) | n | % | Mean count (logCFU $L^{-1}$) | Range (logCFU $L^{-1}$) |
| Hotels not contaminated by Lspp [a] | 4 | 6.3 | - | - | 18 | 28.6 | - | - | 14 | 22.2 | - | - |
| Hotels contaminated by Lspp | 59 | 93.7 | 2.63 | 0.48-5.10 | 45 | 71.4 | 2.84 | 1.32-4.90 | 49 | 77.8 | 2.51 | 0.48-5.60 |
| One single species/serogroup | 20 | 31.7 | 2.6 | 0.48-4.60 | 31 | 49.2 | 2.73 | 1.32-4.90 | 14 | 22.2 | 2.19 | 0.48-3.48 |
| LP1 | 6 | 9.5 | 3.03 | 1.61-4.60 | 10 | 15.9 | 2.78 | 1.32-4.60 | 2 | 3.2 | 2.65 | 2.12-3.15 |
| LP2-14 | 14 | 22.2 | 2.41 | 0.48-4.60 | 21 | 33.3 | 2.71 | 1.32-4.90 | 12 | 19.0 | 2.11 | 0.48-3.48 |
| LnP | 0 | 0.0 | - | - | 0 | 0.0 | - | - | 0 | 0.0 | - | - |
| Several species/serogroup | 39 | 61.9 | 2.62 | 0.48-5.10 | 14 | 22.2 | 2.84 | 1.32-4.85 | 35 | 55.6 | 2.51 | 0.48-5.10 |
| LP1 + LP2-14 | 19 | 30.2 | 2.49 | 0.48-4.73 | 5 | 7.9 | 2.65 | 1.32-4.60 | 19 | 30.2 | 2.5 | 0.48-4.73 |
| LP1 + LnP | 0 | 0.0 | - | - | 2 | 3.2 | 2.81 | 1.32-4.70 | 1 | 1.6 | 2.6 | 2.6 |
| LP2-14 + LnP | 0 | 0.0 | - | - | 3 | 4.8 | 2.82 | 1.32-4.70 | 1 | 1.6 | 1.91 | 1.91 |
| LP1 + LP2-14 + LnP | 20 | 31.7 | 2.74 | 0.48-5.10 | 4 | 6.3 | 3.11 | 1.32-4.85 | 14 | 22.2 | 2.57 | 0.48-5.10 |

[a] Lspp, member of the genus *Legionella*
LP1, *Legionella pneumophila* ser. 1 positives
LP2-14, *Legionella pneumophila* ser. 2-14 positives
LnP, *Legionella* no pneumophila positives
